# PKCε activator protects hippocampal microvascular disruption and memory defect in 3×Tg-Alzheimer’s disease mice with cerebral microinfarcts

**DOI:** 10.3389/fnagi.2023.1272361

**Published:** 2023-12-15

**Authors:** Huaixing Wang, Zongxiu Zhang, Jarin Hongpaisan

**Affiliations:** Department of Medicine, Center for Translational Medicine, Sidney Kimmel Medical College, Thomas Jefferson University, Philadelphia, PA, United States

**Keywords:** cerebrovascular disease, microvessel, hypoxia, oxidative stress, VEGF

## Abstract

**Background:**

Current evidence suggests that microvessel disease is involved in Alzheimer’s disease (AD). Cerebrovascular disease correlates with cardiovascular disease and is complicated in ≈40% of AD patients. The protein kinase C (PKC) ε activator DCPLA can stimulate human antigen (Hu) R that prevents degradation and promotes the translation of mitochondrial Mn-superoxide dismutase (MnSOD) and vascular endothelial growth factor-A (VEGF) mRNAs.

**Methods:**

To induce brain microinfarcts, we injected triple transgenic (3×Tg) and wild-type (WT) control mice with microbeads (20 μm caliber) into common carotid arteries, with or without the DCPLA-ME (methyl-ester) for 2 weeks. After water maze training, mice at 16 months old were examined for confocal immunohistochemistry at a single cell or microvessel level in the hippocampal CA1 area, important for spatial memory storage, and in the dorsal hippocampus by western blots.

**Results:**

In 3×Tg mice without cerebral microinfarcts, an accelerating age-related increase in (mild) oxidative stress and hypoxia inducible factor (HIF)-1α, but a reduction in VEGF, mitochondrial transcription factor A (TFAM), and MnSOD were associated with capillary loss. The change was less pronounced in arterioles. However, in 3×Tg mice with cerebral microinfarcts, increasing arteriolar diameter and their wall cells were related with the strong oxidative DNA damage 8-hydroxy-2′-deoxyguanosine (8-OHdG), apoptosis (cleaved caspase 3), and sustained hypoxia (increased HIF-1α and VEGF/PKCε/extracellular signal regulated kinase or ERK pathway). Microocclusion enhanced the loss of the synaptic marker spinophilin, astrocytic number, and astrocyte-vascular coupling areas and demyelination of axons. DCPLA-ME prevented spatial memory defect; strong oxidative stress-related apoptosis; sustained hypoxia (by reducing HIF-1α and VEGF); and exaggerated cell repair in arteriolar walls, pericapillary space dilation, neuro-glial-vascular disruption, and demyelination.

**Conclusion:**

In conclusion, in 3×Tg mice with cerebral microinfarcts, sustained hypoxia (increased HIF-1α and VEGF signals) is dominant with arteriolar wall thickening, and DCPLA has a protective effect on sustained hypoxia.

## Introduction

Alzheimer’s disease (AD) was previously described as non-vascular dementia (Kalaria and Ballard, 1999; Attems and Jellinger, 2014). Cerebrovascular disease correlates with cardiovascular disease and is complicated in ≈40% of AD patients (Arvanitakis et al., 2011). Dilated perivascular (Virchow–Robin) spaces are another aging marker, which usually manifest as cerebral microvascular (MV) disease, hemorrhage, and learning and memory defect (Kalaria and Ballard, 1999; Kövari et al., 2013). Severe perivascular space enlargement is the indicator for cerebral infarcts (a hallmark of cerebrovascular disease or stroke) (Kalaria and Ballard, 1999; Kövari et al., 2013). Recent evidence suggests that age-related cerebral MV change and cardiovascular disease are strong risk factors for AD (Schneider, 2009; Arvanitakis et al., 2011; Brown and Thore, 2011; Attems and Jellinger, 2014). Cardiovascular disease reduces cerebral blood flow, and hypoperfusion of microvessels can induce microinfarcts in some brain regions (Kemper et al., 1999). An increase in microinfarcts correlates with global decline in cognitive performance, dementia, and AD (Schneider, 2009; Arvanitakis et al., 2011). Microinfarcts, which can be detected under a microscope, were present in 30% of subjects (either cortical, subcortical, or multiple), and 45% of subjects who had microinfarcts did not exhibit macroscopic infarcts (Arvanitakis et al., 2011). Microinfarcts may progress to a vascular lacuna lesion (with a degenerated blood vessel in the middle of the lacuna) or cystic microinfarcts/infarcts, which are visible with the naked eye and present in 30%–60% of AD brains at autopsy (Arvanitakis et al., 2011). Therefore, mild cerebrovascular disease (microinfarcts, etc.) may contribute to and/or is complexed with pathogenesis of AD.

Under normoxia (normal O_2_ level), mitochondrial respiration consumes greater than 90% of the oxygen in humans. The remaining oxygen (~10%) activates prolyl hydroxylase (PHD) that enhances von Hippel–Lindau (pVHL), resulting in the degradation of HIF-1α (Wu et al., 2018; Yeo, 2019; Kim et al., 2020). In response to sustained hypoxia (~0% cytosolic O_2_), the mitochondria consume almost all the oxygen and remove free cytosolic oxygen. In the cytosol, low oxygen inhibits PHD, leading to an increase in HIF1-α stability (Wu et al., 2018; Yeo, 2019; Kim et al., 2020). Formation of HIF-1α and HIF-1β heterodimers activates the transcription of VEGF, VEGF receptor 2 (VEGFR2), and inducible nitric oxide synthase (iNOS) (Ji et al., 2014). These factors participate in the adaptive response to hypoxia by increasing tissue perfusion and oxygenation, thereby aiding in recovery from the initial hypoxic insults.

Aging and hypoxia (~2%–6% cytosolic O_2_) reduce nicotinamide adenosine dinucleotide (NAD^+^) and activity of sirtuin 1 (SIRT1), a NAD^+^-dependent deacetylase, thereby decreasing pVHL-dependent degradation of HIF-1α (Yeo, 2019). Increased HIF-1α inhibits c-Myc and mitochondrial transcription factor A (TFAM), important for the expression of mitochondrial biogenesis and antioxidants (Yeo, 2019). The decrease in c-Myc function reduces VEGF expression (Baudino et al., 2002; Florea et al., 2013). HIF-1α also inhibits peroxisome proliferator-activated receptor-gamma coactivator-1β (PGC-1β) activity, resulting in the downregulation of mitochondrial genes and MnSOD (Lu et al., 2010; Yeo, 2019). These result in oxidative stress and mitochondrial dysfunction (Lu et al., 2010; Bereiter-Hahn, 2014).

The embryonic lethal, abnormal vision, *Drosophila* (ELAV)-like, or Hu family proteins can bind with AU-rich element (ARE) sequences in the 3′-untranslated region (3′-UTR) of mRNA. VEGF and MnSOD mRNAs contain several ARE sequences. PKCε activates HuR and promotes VEGF and MnSOD mRNA stabilization, which enhances their protein synthesis in human brain MV endothelial cells (Millien et al., 2022). Activation of VEGF induces the downstream tyrosine kinase Src/Akt signal pathway and the serine/threonine kinase PKC/extracellular signal-regulated kinase (ERK1/2) signal pathway. Both pathways can activate vascular cell proliferation (Nicolau et al., 2018).

The present study investigated the effect of artificial cerebrovascular disease in triple transgenic (3×Tg) mice injected with microbeads into the right common carotid to induce arteriolar microocclusion (MI) and microinfarcts. We also studied the effect of PKCε-specific activator DCPLA-ME. Spatial learning and memory were determined with water maze training. Using immunohistochemistry and western blots, we studied changes in capillaries and arterioles in the CA1 stratum radiatum of the hippocampus, where synaptogenesis in mushroom-shaped dendritic spines is important for spatial memory (Hongpaisan and Alkon, 2007). We also determined changes in astrocyte-vascular (A-V) contact related to synaptic density and myelinated axons.

## Materials and methods

### Mouse model

We used 23 wild-type (WT) and 35 homozygous transgenic B6;129 male and female 3×Tg mice. 3×Tg mice express three mutations associated with familial AD: APP (amyloid precursor protein) KM670/671NL (Swedish), MAPT (microtubule-associated protein Tau) P301L, and PSEN1 (presenilin-1) M146V (Oddo et al., 2003). All experiments were conducted in accordance with the National Institutes of Health (NIH) Guide for the Care and Use of Laboratory Animals.

### Common carotid artery injection of microbeads

This injection method was previously used for intra-arterial delivery of cell-therapies in a mouse stroke model without ischemic injury or alterations in cerebral blood flow (Silasi et al., 2015). Mice at about 16 months old were anesthetized with 5.0% isoflurane and maintained on 1.5% isoflurane in 70% N_2_O and 30% O_2_ using a capillaries-animal anesthesia system. The local anesthetic bupivacaine (2 mg/kg) was injected subcutaneously at the incision site. The surgical site was shaved with hair removal lotion (Nair^®^, Church and Dwight, Ewing, NJ) and cleaned with 70% ethanol and povidone iodine (Betadine^®^, Avrio Health, Stamford, CT). Before surgery, the depth of anesthesia was assessed by toe pinch. The right common carotid artery was exposed through a neck incision. The external carotid artery and the pterygopalantine arteries were tied with suture. About 2000 Fluoresbrite Yellow Green microspheres at ≈20.0 um caliber (Polysciences, Warrington, PA) suspended in a 100 μL of phosphate buffer saline (PBS) were injected into the common carotid artery over approximately 1 min with a 33G needle. Microspheres traveled towards the right internal carotid artery, the circle of Willis, and then entered the right and left brain. After removing the needle, bleeding was controlled by absorbable suture knot. Unilateral common carotid artery occlusion can reduce blood flow to the brain, similar to aging conditions (Thong-Asa and Tilokskulchai, 2014).

### Drug treatment

About 18 h after surgery, mice were peritoneally injected with methyl ester of 8-[2-(2-pentylcyclopropylmethyl)-cyclopropyl]-octanoic acid (DCPLA-ME, MedChemExpress, Monmouth Junction, NJ) at 3 mg/kg body weight in sterile normal saline (3 times/week). Non-treated groups received the same vehicle volumes, mechanism of delivery, and frequency of administration as the treated groups.

### Water maze training

Eight days after surgery, mice were moved to the test room in their home cages at least 1 h before daily trials. The maze pool had a diameter of 114 cm and height of 60 cm and was filled with 40 cm H_2_O (22 ± 1°C) mixed with 200 mL of non-toxic white Tempera (BesTemp, Certified Color Corp., Orange, CA). The maze was divided into four quadrants. Mice were trained for 5 days (3 trials/day) to find a hidden platform (9 cm diameter) centered in one of the quadrants and submerged about 2 cm below the water surface. At the start of all trials, mice were placed individually in the water facing the maze wall, using different starting positions for each trial, and allowed to swim until they found the platform, where they remained for 20 s before being returned to their home cages. A mouse that failed to find the platform within 1.5 min was guided there by the investigator, with 90 s scored. The swim path was recorded with a video-tracking system, which computed latency to the platform, swim distance, and percentage of time spent in the quadrants. At 24 h after the training trials, a probe trial (a quadrant test or retention trial) was given with the platform removed to assess memory retention for its location by the distance the mouse moved in the quadrants. The video-tracking system tracked the animal’s movements in each quadrant for 1 min.

### Animal brain tissue preparation

Mice were deeply anesthetized with intraperitoneal injection of 100 mg/kg body weight ketamine and 10 mg/kg xylazine. Animals were perfused through the heart with cold PBS for less than 4 min to wash out the blood and subsequently with 4% paraformaldehyde in PBS. Brains were then removed, postfixed for 20 min, and stored in PBS at 4°C. The dorsal hippocampi were sectioned with a cryostat, and 4 hippocampal sections (30 μm thickness) were selected every 400 μm for each hippocampus.

### Immunohistochemistry

The samples were treated with Image-iT FX signal enhancer (Thermo Fisher Scientific, Grand Island, NY, United States) for 30 min at room temperature and then with 5% normal goat serum and 0.5% Triton X-100 in PBS for 50 min to block non-specific protein binding sites. Primary antibodies were: 8-OHdG (mouse monoclonal antibody; 1:100; Genox, Shizuoka, Japan, cat # N45.1); cleaved caspase 3 (rabbit polyclonal IgG; 1:100; Cell Signaling Tech, Danvers, MA, cat # 96645); MnSOD (rabbit; 1:400; MilliporeSigma, Burlington, MA, cat # 06-984); ERK1/2 (mouse; 1:500; Invitrogen, Waltham, MA, cat # 13-6200); HIF-1α (mouse; 1:250; R&D Systems/Bio-Techne, Minneapolis, MN, cat # MAB19351); TFAM (rabbit polyclonal IgG; 1:500; Invitrogen, cat # MA5-35365); VEGF (mouse monoclonal IgG; 1:50; Santa Cruz Biotechnology, cat # sc-7269); PKCε (rabbit; 1:500; MilliporeSigma, cat # 06-991); glial fibrillary acidic protein (GFAP) (rabbit; 1:1,000; Thermo Fisher Scientific, cat # J64334); neurogranin (rabbit; 1:500; MilliporeSigma, cat # AB5620); synaptophysin (mouse; 1:500; MilliporeSigma, cat # MAB5258-I); and myelin basic protein (rabbit, 1:100, Protein Tech, Rosemont, IL, cat # 10458-1-AP) at 4°C for 24 h. Tissue sections were switched to a new incubation solution and washed with PBS (3 times, 5 min each). The samples were then incubated with Alexa Fluor 488 anti-mouse IgG (1:1,000; Thermo Fisher Scientific, cat # A32731) or Alexa Fluor 568 donkey anti-rabbit IgG (1:1,000; Thermo Fisher Scientific, cat # A10042) for 3 h at room temperature. Vascular endothelia were stained at room temperature for 3 h with the DyLight fluor 594-conjugated *Lycopersicon esculentum* (tomato) lectin (Vector Laboratories, Burlingame, CA, 1:50). The sections were mounted using Prolong glass antifade mountant with NucBlue stain (Invitrogen) to counter stain nuclei.

### Confocal microscopy

The images were oriented with a Zeiss Axio Observer Z1 microscope equipped with a 710 confocal scanning system using the 10× objective lens in the DAPI channel (for staining DNA in nuclei). The random area that appeared immediately after switching to the higher magnification lens, 63X Plan-APO Chromat oil immersion objectives (1.4 NA), was imaged for appropriate fluorescence. Confocal images were acquired in line scan mode with a pinhole of approximately 1.00 Airy unit, and averaged data from several (4×) images were reported. Using range (red/blue) indicator in the software, the maximal gray level was set at the under-saturation of each fluorescence channel, except the vascular endothelial cell marker tomato lectin was imaged at saturated intensity because it was not used for quantification. Images were obtained and quantified with the NIH ImageJ program. Under the ImagJ program, the pixel gray levels on the original confocal images were not changed during the adjustment of confocal images on the computer monitor.

Due to thick tissue samples, the insufficiency of passive penetration of antibodies and dyes did not generate uniformly deep staining. Quantification of fluorescence intensities of targeted proteins or nucleoside were therefore normalized with DAPI fluorescence (DNA). Most control data were set at 100%, and other experimental data were defined as percentage of their controls. In the illustrated figures, the brightness and/or contrast of DAPI and other fluorescence channels in an individual image were manually adjusted until DAPI fluorescence among experimental groups reached the same levels, and, presumably, DNA was stable in all nuclei. Because the blood vessel marker tomato lectin was not used for quantification and imaged at saturated intensity, in the figure panels, tomato lectin fluorescence was adjusted separate from adjustments made to DAPI.

### Densities of pre- and postsynaptic structures

After immunohistochemical processing, we measured the densities of presynaptic axonal terminals (synaptophysin grains) and postsynaptic membranes (neurogranin grains) per 33.7 × 33.7 × 0.6 μm^3^ volume. Images were analyzed by particle counting using the NIH ImageJ program commands. The 8-bit gray scale images were inverted so that the dark pixels became light and vice versa. The background of the photographic negative was subtracted with a rolling ball radius at least the size of the largest object that was not part of the background. Using the threshold method, the images were converted from an 8-bit (256 shades of gray) to a 1-bit (black or white) to define an individual pixel as background or particle component. The above procedures were repeated with a different rolling ball radius and threshold method until the particles were the same sizes of synaptophysin or neurogranin particles seen in the original confocal images. Only particles within the range of particle sizes of the measured structures were defined and counted. In addition, synaptopysin intensity was analyzed from the original images (33.7 × 33.7 μm^2^) without the above adjustment, used for grain counting.

### Western blotting

Protein extraction was adapted from Thacker et al. (2021). Fixed-tissue samples were sonicated in 500 μL of modified fixed tissue lysis buffer [containing: 100 mM NaCl, 25 mM EDTA, 500 mM TRIS-HCl, 1% (v/v) Triton X-100, 1% (v/v) IGEPAL CA-630 (NP40), 2% (w/v) SDS, and protease inhibitor cocktail (Thermo Fisher Scientific)]. Homogenates were incubated at 90°C for 120 min under gentle agitation (300 rpm on a shaker) followed by centrifugation (1,000 × *g*) at 4°C. Supernatants were collected, and protein concentrations were determined with a Bio-Rad DC protein assay kit and aliquots stored at −80°C. Proteins were separated on Nu-Page 4%–12% Bis-Tris polyacrylamide gels (Invitrogen). Using an iBlot2 (Thermo Fisher Scientific), proteins were transferred to nitrocellulose membranes. Membranes were incubated with Odyssey blocking buffer (LI-COR) for 1 h at room temperature. Membranes were then treated with primary antibodies against MnSOD (rabbit polyclonal IgG; 1:4,000; MilliporeSigma, cat # 06-984); β-Actin (rabbit polyclonal IgG; 1:25,000; Novus Biological, cat # NBP2-76367); GFAP (rabbit; 1:1,000; Thermo Fisher Scientific, cat # J64334); myelin basic protein (rabbit, 1:2,000, Protein Tech, Rosemont, IL, cat#10458-1-AP); and neurogranin (rabbit; 1:500; MilliporeSigma, cat#AB5620). Blots were then incubated with DyLight 680 anti-mouse antibody (1:1,000, Invitrogen, cat # 35518) and DyLight 800 anti-rabbit antibody (1:1,000, Cell Signaling Technology, cat # 5151). We used a LI-COR imaging system to image the blots. The densitometric value for the protein were quantified with the NIH ImageJ. The target protein normalized with β-actin was used for analysis.

### Statistical analysis

For behavioral and morphological studies, data were analyzed with analysis of variance (ANOVA). Data with a significant overall difference among the groups as demonstrated with an ANOVA analysis were further analyzed for Tukey’s multiple comparison or *t*-test. For western blots, only *t*-test was performed. The confidence level was 95% (*α* = 0.05).

## Results

### Cerebrovascular microocclusion induces cerebral microinfarcts

**Figure 1:**
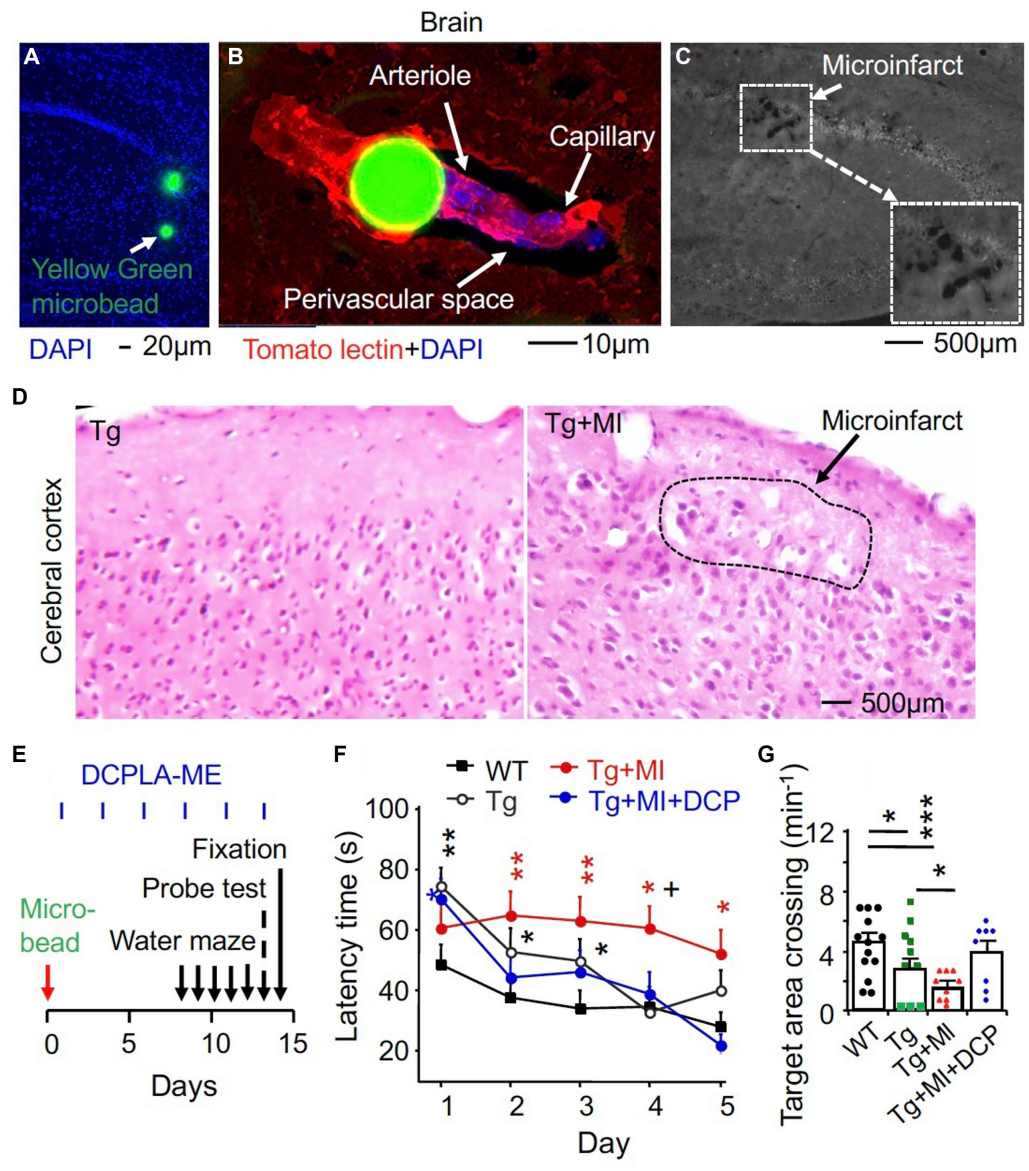
The PKCε activator DCPLA-ME improves spatial learning and memory defect in 3×Tg mice complexed with microocclusion-induced microinfarct. Injection of 2,000 yellow-green plastic microbeads into the right common carotid artery was performed to induce microinfarct (Silasi et al., 2015). **(A)** Via the Circle of Willis, microbeads at 20 μm diameter entered into the whole brain. **(B)** Microbeads impeded microcirculation (blood flow into arterioles and then capillaries). **(C)** A microinfarct induced by cerebrovascular microocclusion (MI) in several brain regions. **(D)** Hematoxylin and eosin staining showing microinfarcts. **(E)** Schematic drawing summarized time schedule of experiment. At 24 h after microbead injection, DCPLA-ME treatment was started (i.p., 3 times/week). **(F)** Learning acquisition: at 1 week after microbead injection, we trained 3×Tg transgenic (Tg) and wild-type (WT) mice in a water maze pool for 5 days (3 swims/day). The escape latency time to find the hidden platform was quantified. **(G)** Memory retention was assessed with a probe test that allowed mice to find the target area of the removed platform at 24 h after 5 days of water maze training. Data dots (panel **F**) and bars (panel **G**) were means ± SEM from *n* = 15–21 swims per days from 5–7 mice per group or *n* = 8–14 probe test from 8–14 mice per group. Each dot blot on graph bar in panel E was an individual animal mean. ^*^p = 0.05, ^**^p = 0.01, ^***^p = 0.001, and ^+^*p* = 0.05, compared with Tg mice. In panel **(E)**, asterisk(s) over the data is/are compared with WT mice; and in panel **(F)**, asterisks over a line are compared with those 2 data bars.

Mice were injected into right common carotid artery after neck surgery with about 2,000 yellow green microbeads at ≈20.0 um diameter (Silasi et al., 2015). Microbeads traveled through the Circle of Willis and entered both the left and right brains. The microbeads at ≈20.0 um diameter impeded local microcirculation through terminal arterioles (>6 μm in diameter; Figure 1B) (McDowell et al., 2021). Microbeads were small enough to pass cerebral arteries throughout the whole brain (Figure 1A). This resulted in microinfarct (artificial cerebrovascular disease) (Figures 1C,D).

### The PKCε activator DCPLA-ME improves spatial learning and memory defect in 3×Tg mice with microocclusion

We recently demonstrated that the PKCε activators can promote VEGF and MnSOD expression and prevent MV loss and/or spatial memory defect in aged rats and Tg2567 mouse model of AD (Millien et al., 2022). Tg2576 mice overexpressed only Amyloid beta (Aβ) peptide (Westerman et al., 2002). We then aimed to further study therapeutic effect of the PKCε activator DCPLA-ME in 3×Tg mice that expressed both amyloid plaques and neurofibrillary tangles (Oddo et al., 2003) with and without cerebral microinfarcts. After microbead injection, mice were treated with or without the PKCε activator DCPLA-ME. For spatial learning and memory studies, mice were trained to find a submerged platform in a water maze pool (Figure 1E). The training regimen (3 swims per day) was intentionally made more difficult to better reveal cognitive deficits. At 24 h after 5 days of water maze training and learning evaluation, memory retention was assessed with a probe test that allowed mice to find the target area of the removed platform. There were significant differences among all experimental groups for 5 days swim learning (*F*_4,449_ = 8.763, *p* = 0.001, ANOVA) and memory (*F*_4,46_ = 3.379, *p* = 0.017). Compared with WT mice, 3×Tg mice showed a significant impairment in learning (an increase in latency time to reach platform, *p* = 0.002) and memory (to concentrate on the removed platform area, *p* = 0.013) (Figures 1F,G). MI enhanced the learning impairment (*p* = 0.040) and memory defect (*p* = 0.047, Figures 1F,G).

### Microocclusion increases capillary density, arteriolar cells and diameter, pericapillary space enlargement in 3×Tg mouse hippocampal CA1 stratum radiatum

**Figure 2:**
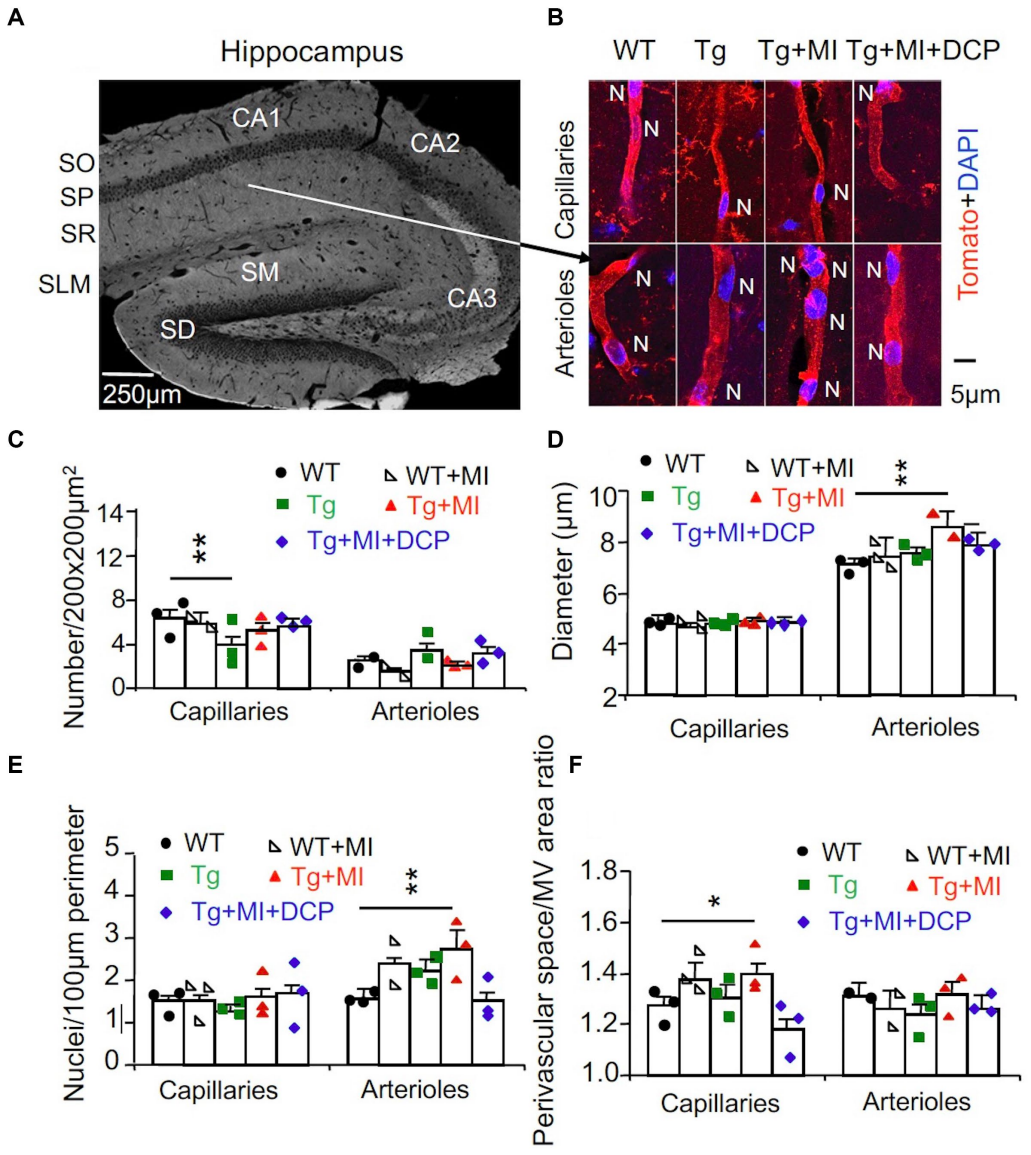
Cerebrovascular occlusion-induced endothelial cell dysplasia of arterioles and/or venules and pericapillary space dilation in 3×Tg mice hippocampi are prevented with the DCPLA-ME. 3×Tg (Tg) Mice were injected with microbeads into the right common carotid to induce arteriolar microocclusion (MI) and microinfarcts in the brains and/or with the PKCε-specific activator DCPLA-ME treatment, compared to non-treated 3×Tg and wild-type (WT) mice. **(A)** After spatial learning and memory studies with water maze training, mice at 16 months old were used for morphometry of capillaries (<6 μm in diameter) and arterioles (>6 μm in diameter) (McDowell et al., 2021) in the hippocampal CA1 stratum radiatum (SR). SO, stratum oriens; SP, stratum pyramidale; SLM, stratum lacunosum-moleculare; SM, stratum moleculare; SG, stratum granulosum. **(B)** Microvessels were stained with the vascular endothelial cell marker tomato lectin (red), and nuclei were stained with DAPI (blue). **(C)** The microvascular (MV) number per 200 × 200 μm^2^ of hippocampal CA1 area. **(D)** The MV diameter. **(E)** The number of nuclei stained with DAPI (blue) per 100 μm perimeter of microvessels. **(F)** The ratio of perivascular space divided by the MV area. Data bars were mean ± SE from *n* = 34–74 areas or *n* = 60–242 capillaries, 33–80 arterioles from 3 mice per group. Each dot blot on graph bar was an individual animal mean. ^*^p < 0.05 and ^**^p < 0.01. Asterisk(s) over a line are compared with those 2 data bars.

At 24 h after the probe test, morphological change in capillaries and arterioles were further studied *in situ*. Mice were fixed with formaldehyde, and brains were used for histochemical and microscopic studies (Figure 2A). Vascular endothelial cells were stained with tomato lectin and imaged with confocal microscope (Figure 2B). We quantified the change in capillaries (<6 μm in diameter) and arterioles (>6 μm in diameter) (McDowell et al., 2021). ANOVA revealed significant differences among animal groups for the capillary number (*F*_4,294_ = 3.321, *p* = 0.011) and perivascular space (*F*_4,282_ = 5.213, *p* = 0.001) as well as for the arteriolar diameter (*F*_4,289_ = 2.547, *p* = 0.040) and endothelial cell density (*F*_4,153_ = 3.120, *p* = 0.017) (Figures 2C–F). For the post-hoc test, Tukey multiple comparison was performed.

In WT mice, MI did not affect the number of capillaries and arterioles per 200 × 200 μm^2^ hippocampal area, diameter, endothelial cell number/100 μm perimeter, or the ratio of perivascular space divided by MV area of capillaries and arterioles (Figures 2C–F, compared WT + MI to WT). In 3×Tg mice, the number of capillaries, but not arterioles, decreased (*p* = 0.015) (Figure 2C, Tg vs. WT). In 3×Tg + MI mice, the density of capillaries was not different from WT and 3×Tg mice (Figure 2C). This suggests that MI increases angiogenesis in 3×Tg mice. The data showed that MV diameters in the hippocampal CA1 areas were smaller than the microbeads (20 μm in diameter). Most capillaries were in the range of 3–5 μm in diameter. Arterioles were mostly 7–10 μm; less than 5% were 10–20 μm in diameter. MI increased the diameter of arterioles (*p* = 0.037) and the number of their nuclei (*p* = 0.030) (Figures 2D,E; Tg + MI vs. Tg). The increased nuclei were the large oval, round, and spindle shape of endothelial cells rather than small and thin nuclei of pericytes and smooth muscle cells (Figure 2B). MI increased (*p* = 0.001) the ratio of perivascular space divided with its MV area for capillaries but not arterioles (Figure 2F).

### The PKCε-specific activator DCPLA-ME prevents learning and memory defects, arteriolar alteration, and pericapillary space dilation In hippocampal CA1 area of 3×Tg mouse with microocclusion

DCPLA-ME treatment protected the learning (*p* = 0.002) and memory defect (*p* = 0.039) in 3×Tg mice with MI (Figures 1F,G; Tg + MI + DCP vs. Tg + MI). DCPLA-ME also prevented an increase in the diameter or wall cells of arterioles (*p* = 0.025) and the pericapillary space dilation (*p* = 0.002) in 3×Tg mice with MI (Figures 2D–F). The results indicate that DCPLA-ME prevents the effect of cerebrovascular MI.

### Capillary loss in hippocampal CA1 area is related to a decrease in mitochondrial MnSOD in hippocampal CA1 area of 3×Tg mice (without microocclusion)

**Figure 3:**
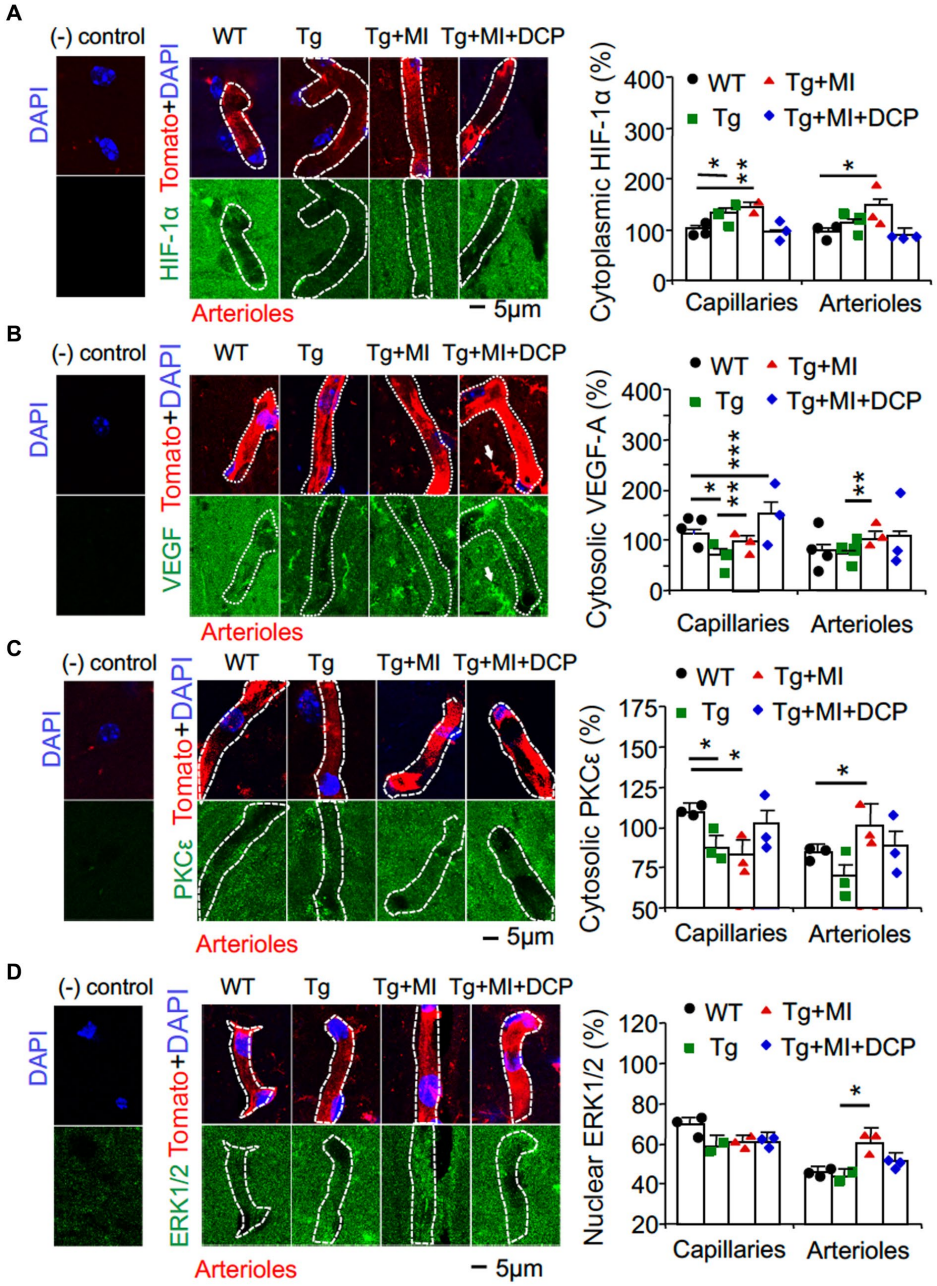
Cerebral microocclusion increases HIF-1α and VEGF in capillaries and arterioles, which is prevented with DCPLA-ME, in hippocampal CA1 area of 3×Tg. 3×Tg (Tg) mice were injected with microbeads into the right common carotid to induce arteriolar microocclusion (MI) and microinfarcts in the brains and/or with the PKCε-specific activator DCPLA-ME treatment, compared to non-treated 3×Tg and wild-type (WT) mice. After water maze training, double immunohistochemistry of the vascular endothelial cell marker tomato lectin was used to investigate change in **(A)** HIF-1α, **(B)** VEGF, **(C)** PKCε, and **(D)** ERK1/2. In confocal image panels, negative (−) controls were immunohistochemistry without primary antibody and tomato lectin. Compared to negative controls, confocal images showed that HIF-1α, VEGF, PKCε, and ERK1/2 were not specifically expressed in capillaries (<6 μm in diameter) and arterioles (>6 μm in diameter) (McDowell et al., 2021). **(B)** White arrows pointed to microglia that were also stained with tomato lectin and expressed VEGF. The small profiles around arterioles were microglia, also stained with tomato lectin. Data bars were mean ± SE from *n* = 30–67 microvascular (MV) cells from 3–5 mice per group. Each dot blot on the graph bar was an individual animal mean. ^*^p < 0.05, ^**^p < 0.01, and ^***^p < 0.001. Asterisk(s) over a line are compared with those 2 data bars.

**Figure 4:**
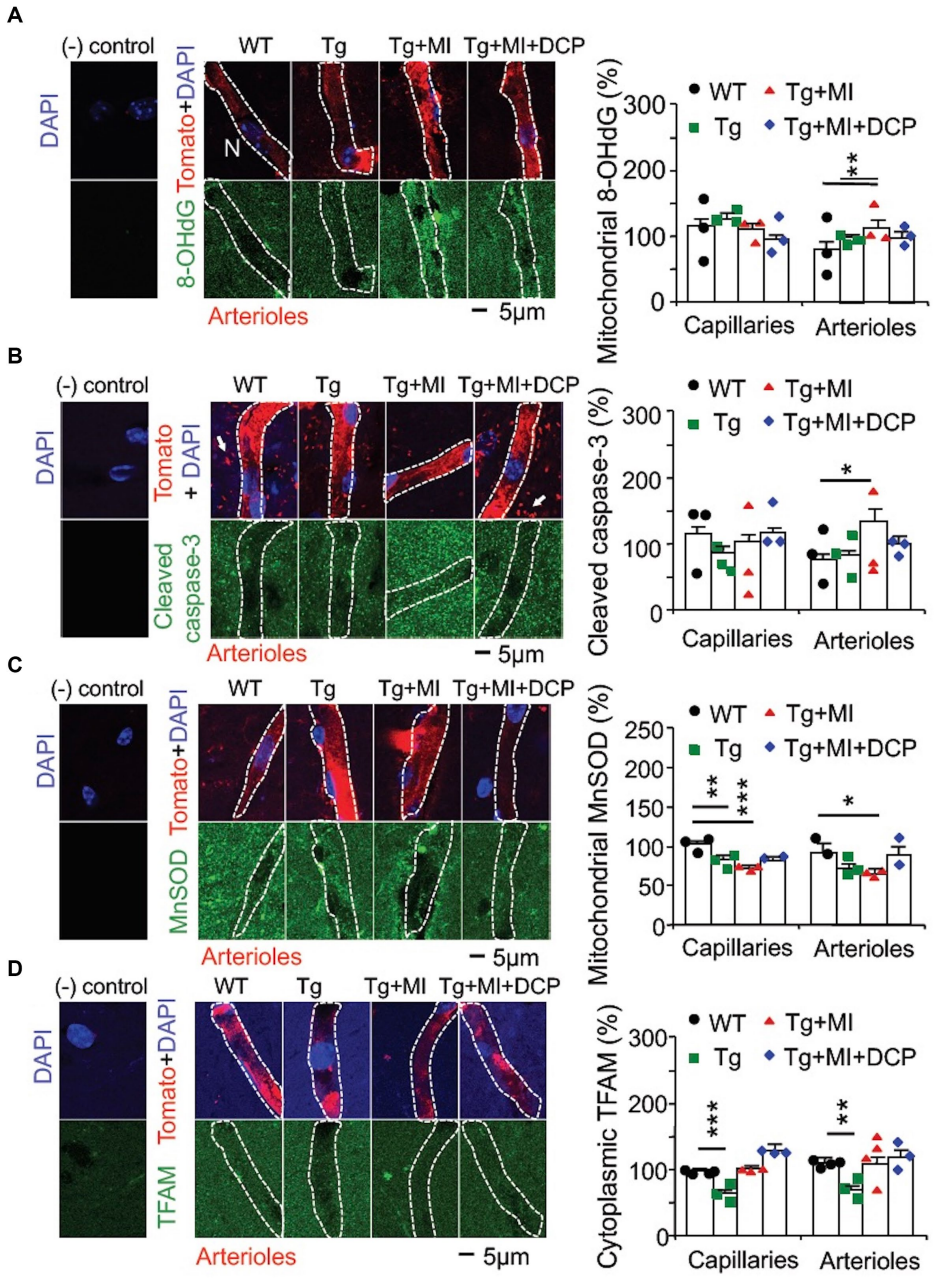
Cerebral microocclusion (MI) induces strong oxidative stress-related apoptosis in arterioles and increases mitochondrial transcription in both capillaries and arterioles that are prevented with DCPLA-ME in hippocampal CA1 area of 3×Tg mice. 3×Tg (Tg) mice were injected with microbeads into the right common carotid to induce arteriolar MI and microinfarcts in the brains and/or with the PKCε-specific activator DCPLA-ME treatment, compared to non-treated 3×Tg and wild-type (WT) mice. After water maze training, double immunohistochemistry of the vascular endothelial cell marker tomato lectin in hippocampal CA1 stratum radiatum and primary antibody was used to investigate change in **(A)** the oxidative DNA damage marker 8-hydroxy-2′-deoxyguanosine (8-OHdG) in the cytoplasm, presumably mitochondrial DNA damage; **(B)** cleaved caspase-3 (the apoptosis marker); **(C)** mitochondrial MnSOD; and **(D)** TFAM. In confocal image panels, negative (−) controls were immunohistochemistry without primary antibody and tomato lectin. Compared to negative controls, confocal images showed that 8-OHdG, cleaved caspase 3, MnSOD, and TFAM were not specifically expressed in capillaries (<6 μm in diameter) and arterioles (>6 μm in diameter) (McDowell et al., 2021). **(B)** The small profiles around arterioles are microglia (white arrows) that were also stained with tomato lectin. Data bars were mean ± SE from *n* = 19–57 microvascular (MV) cells from 3–4 mice per group. Each dot blot on the graph bar was an individual animal mean. ^*^p < 0.05, ^**^p < 0.01, and ^***^p < 0.001. Asterisk(s) over the data bar is/are compared with WT.

It is well known that the targeted cell signals in the present study are not specially expressed in capillaries and arterioles. VEGF is also expressed in neurons, astrocytes, microglia etc., (Rosenstein et al., 2010; Argaw et al., 2012; Okabe et al., 2020). Therefore, double immunohistochemistry at an individual capillary or arteriole was used (Figures 3, 4). Negative controls (without primary antibody and tomato lectin) for immunohistochemical staining showed that all immunohistochemistry of target cell signal molecules (Figures 3, 4) were not specifically located in capillaries or arterioles.

Oxidative stress is involved in capillary loss as well as age-related arteriolar and artery wall thickening (Jia et al., 2019; Sharma et al., 2022; Wang Y. et al., 2023). We used immunohistochemistry to study the pathogenesis of AD complexed with mild cerebrovascular disease in microvessels double labelled with the vascular endothelial marker tomato lectin at single endothelial cell level. The interaction of hydroxyl radical (HO), the most toxic reactive oxygen species (ROS), with a nucleoside, such as deoxyguanosine, leads to the formation of 8-OHdG (Kasai, 1997). We also investigated the change in cleaved caspase-3 involved in apoptosis and change in mitochondrial MnSOD that affects oxidative stress.

ANOVA revealed a significant difference among animal groups for cytoplasmic (presumably mitochondrial) 8-OHdG (*F*_3,171_ = 3.663, *p* = 0.014) and cytosolic cleaved caspase-3 (*F*_3,123_ = 4.037, *p* = 0.009) in arterioles, but not capillaries, and for mitochondrial MnSOD in both capillaries (*F*_4,171_ = 3.663, *p* = 0.014) and arterioles (*F*_3,102_ = 4.656, *p* = 0.004) (Figures 4A–C). In 3×Tg mice, MnSOD decreased (*p* = 0.002) in capillaries, but not arterioles, while 8-OHdG and cleaved caspase-3 were not affected in both capillaries and arterioles (Figures 4A–C, Tg vs. WT). The data indicates that capillary loss is associated with mild oxidative stress induces in 3×Tg mice.

### Capillary loss is associated with an increase in HIF-1α but reduction of TFAM and VEGF In 3×Tg hippocampal CA1 area

Although the capillary loss is not further changed, the hypoperfusion and hypoxia are promoted during early AD (Brown and Thore, 2011; Hunter et al., 2012). Hypoxia increases HIF-1α but inhibits the mitochondrial transcription marker (TFAM), mitochondrial MnSOD, and VEGF expression (Lu et al., 2010; Yeo, 2019). Therefore, we studied the change in HIF-1α stability and TFAM expression (Figures 3A, 4D; Tg vs. WT). Significant differences among animal groups were observed for HIF-1α in both capillaries (*F*_3,113_ = 4.230, *p* = 0.007) and arterioles (*F*_3,113_ = 3.067, *p* = 0.031) and for TFAM in both capillaries (*F*_3,161_ = 13.064, *p* = 0.001) and arterioles (*F*_3,93_ = 8.592, *p* = 0.001). The post-hoc Tukey multiple comparison exhibited an increase in HIF-1α stability (*p* = 0.018) but a decrease in TFAM expression (*p* = 0.047) in capillaries of 3×Tg mice (Figures 3A, 4D; Tg vs. WT). We also studied the change in VEGF/PKCε/ERK signal cascade. For capillaries, we observed significant differences among animal groups for VEGF (*F*_3,113_ = 4.230, *p* = 0.007) and PKCε (*F*_3,152_ = 2.704, *p* = 0.048), but not ERK (Figures 3B–D). Post-hoc Tukey multiple comparison revealed that VEGF (*p* = 0.012) and PKCε (*p* = 0.030) decreased in capillaries (Figures 3B–D; Tg vs. WT).

The data indicate that capillary loss is related with mild oxidative stress; an increase in HIF-1α; and a decrease in TFAM, VEGF, and PKCε in the hippocampi of 3×Tg mice at 16 months old.

### A decrease in mitochondrial transcription factor A in arterioles of 3×Tg hippocampal CA1 area

In arterioles of 3×Tg mice, HIF-1α stability did not change, but TFAM expression decreased (*p* = 0.005) (Figures 3A, 4D; Tg vs. WT). This indicates a HIF-1α-independent decrease in TFAM in arterioles in the 3×Tg hippocampus. We also studied change in the VEGF/ PKCε/ERK1/2 on cell proliferation in the arterioles and observed significant differences among animal groups for VEGF (*F*_3,113_ = 4.230, *p* = 0.007), PKCε (*F*_3,106_ = 3.755, *p* = 0.014), and ERK1/2 (*F*_3,114_ = 2.858, *p* = 0.04). However, the expression of VEGF, PKCε, and ERK1/2 in arterioles did not change in 3×Tg mice (Figures 3B–D; Tg vs. WT). These results suggest that although the arteriolar structure did not change, the function may be affected due to dysregulation of mitochondrial transcription.

### Cerebral microocclusion induces strong oxidative stress and apoptosis in arterioles, but not capillaries, in 3×Tg mouse hippocampal CA1 area

Cerebrovascular MI increased the strong oxidative DNA damage marker 8-OHdG (*p* = 0.007) and apoptosis (*p* = 0.022), but decreased (*p* = 0.016) mitochondrial MnSOD in arterioles (Figures 4A–C; Tg + Mi vs. Tg). However, in capillaries, cerebrovascular MI reduced MnSOD but did not affect 8-OHdG and cleaved case-3 (Figures 4A–C). These results suggest that MI induces strong oxidative stress and apoptosis in arterioles, while capillary loss in 3×Tg mice with and without MI is related to mild oxidative stress that is not strong enough to induce mitochondrial oxidative DNA damage and apoptosis.

### Cerebral microocclusion increases HIF-1α, TFAM, and VEGF in both capillary and arteriolar cells of 3×Tg hippocampal CA1 stratum radiatum

In 3×Tg mice with MI, HIF-1α stability increased in capillaries (*p* = 0.005) and arterioles (*p* = 0.019). MI increased mitochondrial TFAM in 3×Tg mice to a level not different from WT controls (Figures 3A, 4D; Tg + Mi vs. Tg). VEGF increased (*p* = 0.002) in both capillaries and arterioles of 3×Tg + microinfarcts compared with 3×Tg (Figure 3B). However, MI increased the downstream VEGF molecular target PKCε (*p* = 0.007) and ERK1/2 (*p* = 0.043) in arterioles but not capillaries (Figures 3C,D).

These results indicate that MI induces strong oxidative stress-associated apoptosis and sustained hypoxia (very low O_2_ in the cytoplasm, as mentioned in the Introduction). The sustained hypoxia increases VEGF and its downstream ERK1/2 involved in anti-apoptosis and cell proliferation, resulting in over-proliferation of MV cells and the increased diameter of arterioles.

### DCPLA-ME reduces HIF-1α stability but increases PKCε, VEGF, and MnSOD in hippocampal CA1 capillaries of 3×Tg mice with cerebral microocclusion

When we compared 3×Tg + MI mice with and without DCPLA-ME treatment, DCPLA-ME prevented an increase in HIF-1α (Figure 3A) and prevented the loss of PKCε and MnSOD (Figures 3C, 4C). DCPLA-ME also protected the reduction of VEGF and enhanced (*p* = 0.001) VEGF more than the WT controls (Figure 3B). These results suggest that DCPLA-ME increases PKCε, VEGF (cell proliferation), and MnSOD. An increase in MnSOD protects against oxidative stress and the HIF-1α stability in capillaries of 3×Tg mice with MI.

### DCPLA-ME prevents strong oxidative stress, apoptosis, and increased HIF-1α/VEGF/ERK signal pathway in arterioles of 3×Tg hippocampal CA1 area with cerebral microocclusion

DCPLA-ME prevented strong oxidative DNA damage (8-OHdG), apoptosis (cleaved caspase 3), HIF-1α, VEGF, PKCε, and ERK1/2 in arterioles (Figures 4A,B, 3A–D; Tg + MI + DCP vs. Tg + MI). These results suggest that DCPLA-ME activates anti-apoptosis and prevents exaggerated cell proliferation in arterioles.

### DCPLA-ME protects an upregulation of MnSOD in the dorsal hippocampus of 3×Tg mice with cerebral microocclusion

**Figure 5:**
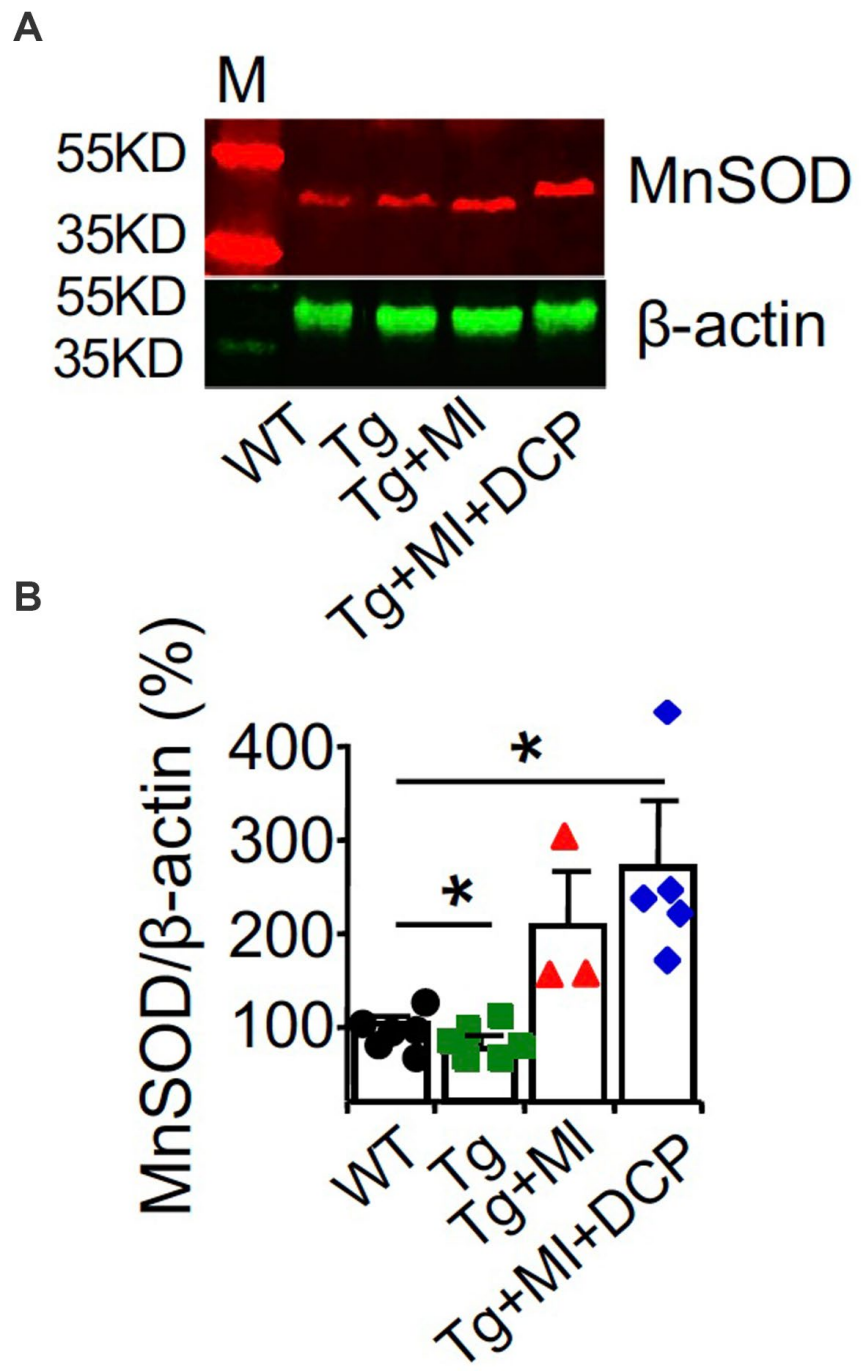
DCPLA-ME prevents an increase in MnSOD in dorsal hippocampus of 3×Tg mouse with cerebral microocclusion. 3×Tg (Tg) mice were injected with microbeads into the right common carotid to induce arteriolar microocclusion (MI) and microinfarcts in the brains and/or with the PKCε-specific activator DCPLA-ME treatment, compared to non-treated 3×Tg and wild-type (WT) mice. After water maze training, dorsal hippocampi were used for **(A)** western blot analysis for **(B)** MnSOD. M, marker for molecular weight. Data bars were mean ± SE from *n* = 3–6 mice per group. Each dot blot on the graph bar was an individual animal mean. ^*^p < 0.05 and ^**^p < 0.01. Asterisk(s) over a line are compared with those 2 data bars.

Western blots of the dorsal hippocampus revealed a decrease in MnSOD (*p* = 0.040) in 3×Tg mouse hippocampus (Figures 5A,B; Tg vs. WT), confirming an age-related change in AD. However, western blots showed an increase in MnSOD (*p* = 0.041) in 3×Tg mouse hippocampus with microinfarcts (Figures 5A,B; Tg + MI vs. Tg), confirming that MI induces sustained hypoxia in 3×Tg mouse hippocampus. DCPLA prevented the rise of MnSOD in 3×Tg mice with cerebral microinfarcts (Figures 5A,B; Tg + MI + DCP vs. Tg + MI).

The results from western blots of MnSOD at the dorsal hippocampal levels (Figures 5A,B) were different from those studied with immunohistochemistry (Figure 4C). This confirms that for targeted cell signals that were not expressed only in blood vessels, immunohistochemistry at an individual capillary or arteriole will give more accurate results than western blot analysis.

### DCPLA-ME prevents the loss of astrocytes and astrocyte-vascular coupling in the hippocampal CA1 area of 3×Tg mice with cerebral microocclusion

**Figure 6:**
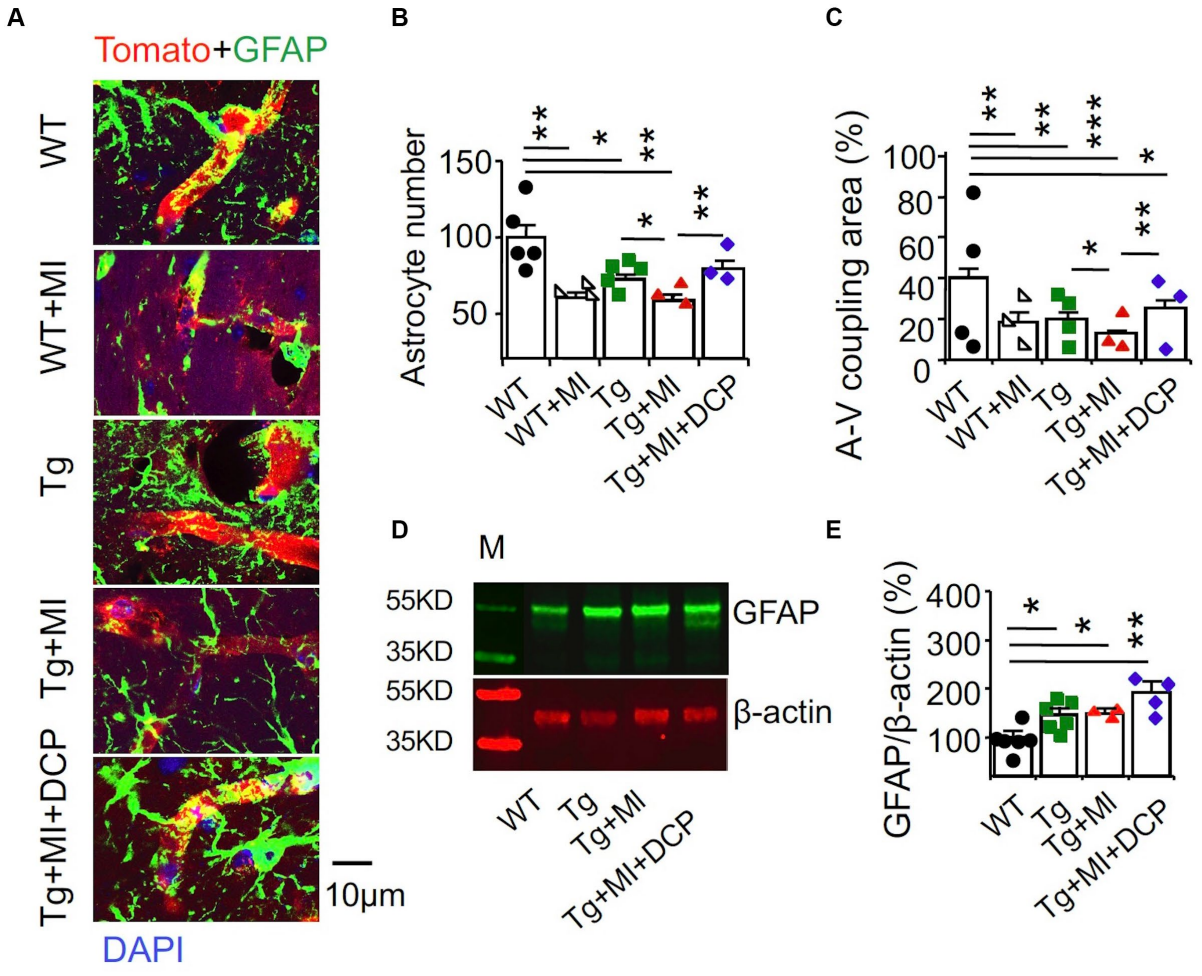
Accelerated disruption of astrocyte-vascular (A-V) coupling is prevented with DCPLA-ME in the hippocampus of 3×Tg-AD mice with microinfarcts. 3×Tg (Tg) mice were injected with microbeads into the right common carotid to induce arteriolar microocclusion (MI) and microinfarcts in the brains and/or with the PKCε-specific activator DCPLA-ME treatment, compared to non-treated 3×Tg and wild-type (WT) mice. After water maze training, mice at 16 months old were used for histology. **(A)** Double immunohistochemistry of the glial fibrillary acidic protein (GFAP) marker for astrocytes (green) and the vascular endothelial cell marker tomato lectin (red) were to determine **(B)** astrocytic number per 135 μm × 135 μm area and **(C)** A-V coupling areas [yellow = colocalization of GFAP (green) and tomato lectin (red)]. **(D)** Western blots of dorsal hippocampus for **(E)** GFAP. M, marker for molecular weight. Data bars were mean ± SE from *n* = 25–56 microvessels or 12–20 areas from 3–4 mice per group or *n* = 3–6 mice per western blot group. Each dot blot on the graph bar was an individual animal mean. ^*^p < 0.05, ^**^p < 0.01, and ^***^p < 0.001. Asterisk(s) over a line are compared with those 2 data bars.

Astrocytes are ideally positioned to mediate neurovascular coupling, relaying signals from neurons to blood vessels that regulate blood flow in the brain (MacVicar and Newman, 2015). Immunohistochemistry of the astrocyte marker GFAP and the vascular endothelial cell marker tomato lectin was performed. In Figure 6A, A-V coupling areas were identified as colocalization (yellow) of astrocytic end feet (green fluorescence) and vascular endothelial cells (red fluorescence). ANOVA showed significant differences among animal groups for the number of astrocytes (*F*_4,72_ = 6.587, *p* = 0.002) and A-V coupling areas (*F*_4,178_ = 8.247, *p* = 0.001). In WT mice, although MI did not induce change in morphometry of microvessels (Figures 2B–E), MI induced loss of astrocytes (*p* = 0.004) and reduction of A-V coupling areas (*p* = 0.003) (Figures 6B,C; WT + MI vs. WT). In 3×Tg mice without MI, we observed the loss of astrocytes (*p* = 0.019) and A-V coupling areas (*p* = 0.001) (Figures 6B,C; Tg vs. WT). MI enhanced the loss of astrocytes (*p* = 0.011) and A-V coupling areas (*p* = 0.042) (Figures 6B,C; Tg + MI vs. Tg). Western blots of the dorsal hippocampus showed an increase in GFAP protein in 3×Tg (*p* = 0.042), 3×Tg + MI (*p* = 0.023), and 3×Tg + MI + DCP mice (*p* = 0.042) (Figures 6D,E). This suggests that the remaining astrocytes are enlarged (hypertrophy) to compensate for the loss of astrocytes. DCPLA-ME prevented the enhancement effect of MI on the loss of astrocytes (*p* = 0.006) and A-V coupling areas (*p* = 0.009) (Figures 6B,C).

### DCPLA-ME prevents synaptic loss in the hippocampus of 3×Tg mice with cerebral microocclusion

**Figure 7:**
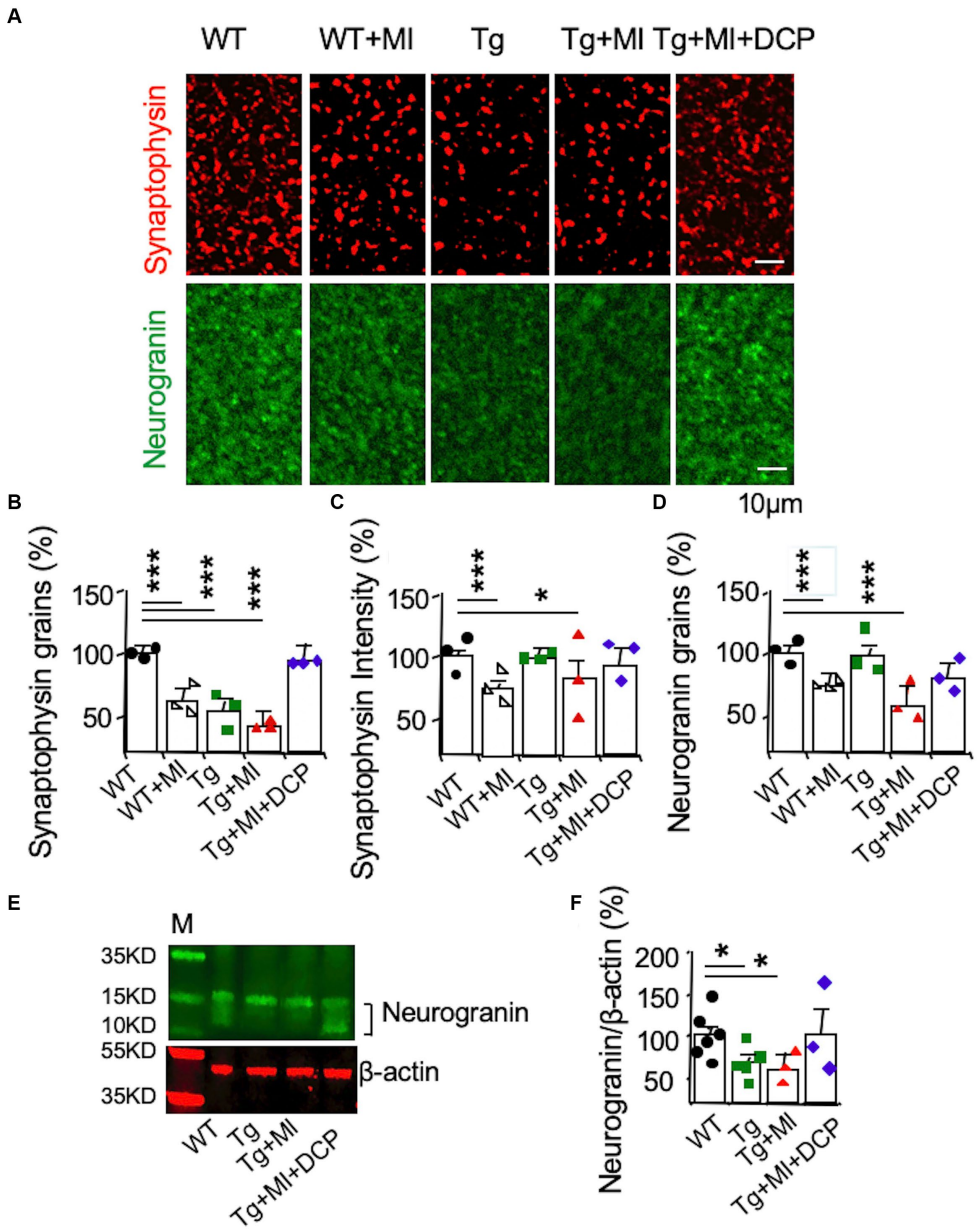
Accelerated synaptic loss is prevented with DCPLA-ME in the hippocampal CA1 of 3×Tg-AD mice with microinfarcts. 3×Tg (Tg) mice were injected with microbeads into the right common carotid to induce arteriolar microocclusion (MI) and microinfarcts in the brains and/or with the PKCε-specific activator DCPLA-ME treatment, compared to non-treated 3×Tg and wild-type (WT) mice. After water maze training, mice at 16 months old were used for histology. **(A)** Immunohistochemistry of the presynaptic vesicle membrane protein synaptophysin (syn) and the postsynaptic membrane protein neurogranin (NG). **(B)** Presynaptic axonal boutons (synaptophysin grains) per 33.7 × 33.7 × 0.6 μm^3^ volume. **(C)** Presynaptic vesicle concentration in axonal boutons (synaptophysin intensity). **(D)** The postsynaptic membranes (neurogranin grains) per 33.7 × 33.7 × 0.6 μm^3^ volume. **(E)** Western blots of dorsal hippocampus for **(F)** neurogranin, M, marker for molecular weight. Data (mean ± SE) from *n* = 18–22 areas from 3–4 mice per group or *n* = 3–6 mice per western blot group. Each dot blot on the graph bar was an individual animal mean. ^*^p < 0.05, ^**^p < 0.01, and ^***^p < 0.001. Asterisk(s) over a line are compared with those 2 data bars.

Next, the effects of changes in capillaries and arterioles as well as A-V coupling on neurons were further investigated. We studied changes in synapses associated with MV damages in 3×Tg mice with MI. Immunohistochemical detection visualized with a confocal microscope (Figure 7A) was used to stain presynaptic axonal boutons (presynaptic vesicle membrane protein synaptophysin) and post-synaptic membranes (neurogranin). Presynaptic axon boutons (synaptophysin grains) and post-synaptic membranes (neurogranin grains) were counted in a 30 μm × 30 μm area. Synaptophysin intensity indicated presynaptic vesicle amount within the axonal boutons. Significant differences among animal groups were observed for presynaptic axon bouton density (*F*_4,95_ = 16.285, *p* = 0.001), presynaptic vesicle concentration (*F*_4,98_ = 6.470, *p* = 0.001), and post-synaptic membrane density (*F*_4,79_ = 8.677, *p* = 0.001).

The number of presynaptic boutons decreased (*p* = 0.001) in 3×Tg mice (Figure 7B; Tg vs. WT). In Figure 7B, although MI induced the loss of presynaptic boutons in WT mice (WT + MI vs. WT), MI did not enhance the loss of presynaptic boutons in 3×Tg mice (Tg + MI vs. Tg). The presynaptic vesicle concentration in 3×Tg mice was not different from that in WT mice (Figure 7C; Tg vs. WT). In Figure 7C, MI induced the loss of presynaptic vesicle concentration in both WT (*p* = 0.001, WT + MI vs. WT) and 3×Tg (*p* = 0.049, Tg + MI vs. Tg) mice. Similar to changes in presynaptic vesicle concentration (Figure 7C), MI activated the loss (*p* = 0.001) of postsynaptic membrane density (neurogranin grains) in WT and 3×Tg mice (Figure 7D). In 3×Tg mice, although the number of postsynaptic membranes did not change, western blots demonstrated a down-regulation of the postsynaptic membrane protein neurogranin (Figures 7E,F). These data suggest the shrinkage of postsynaptic membrane size in 3×Tg mice. DCPLA-ME prevented the reduction of presynaptic vesicles and membranes and postsynaptic membranes from 3×Tg mice with cerebral MI (Figures 7B–F).

### DCPLA-ME rescues the demyelination of axons in 3×Tg hippocampus

The presence and extent of white matter hyperintensities or leukoaraiosis is a radiographic marker (e.g., MRI) of capillaries’ cerebral vessel disease, cognitive impairment, and functional disability (Capizzano et al., 2004). In AD brains, more myelin loss occurs in the late Braak stage (Ihara et al., 2010; Erten-Lyons et al., 2013). Changes in the myelinated axons in the white matter were quantified in the perforated path *that* provides a connectional route from the entorhinal cortex to all fields of the hippocampal formation.

**Figure 8:**
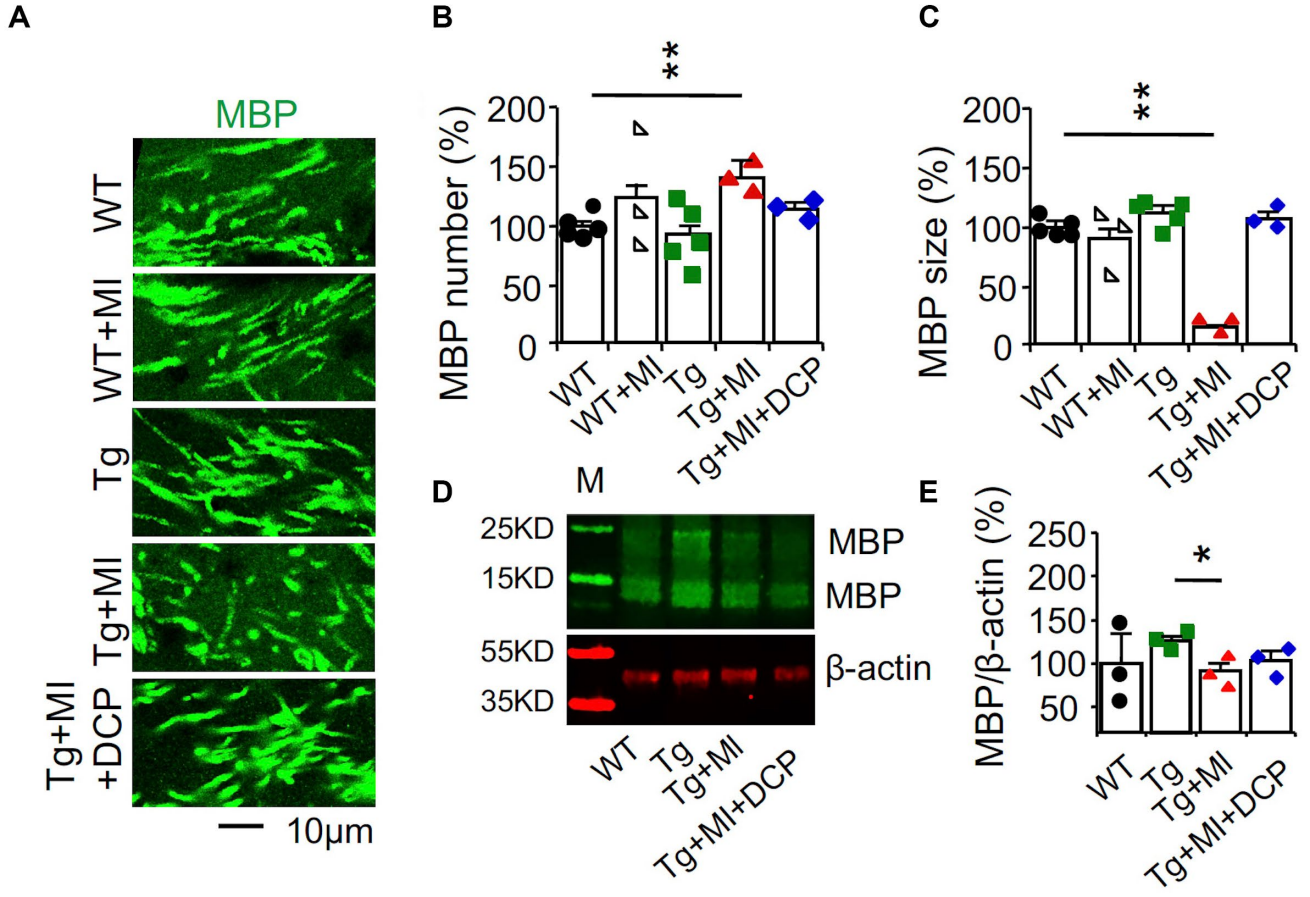
Demyelination is prevented with DCPLA-ME in the hippocampus of 3×Tg-AD mice with microinfarcts. 3×Tg (Tg) mice were injected with microbeads into the right common carotid to induce arteriolar microocclusion (MI) and microinfarcts in the brains and/or with the PKCε-specific activator DCPLA-ME treatment, compared to non-treated 3×Tg and wild-type (WT) mice. After water maze training, mice at 16 months old were used for histology. **(A)** Immunohistochemistry of myelin basic protein (MBP) in the hippocampal CA1 area was performed to display **(B)** the increase in the number of myelinated axons but **(C)** decrease in the size of myelinated axons, suggesting the uneven detachment of myelinated sheath. **(D)** Western blots of dorsal hippocampus for **(E)** neurogranin. M, marker for molecular weight. Data (mean ± SE) from *n* = 12–20 areas from 3–4 mice per group or *n* = 3–6 mice per western blot group. Each dot blot on the graph bar was an individual animal mean. ^*^p < 0.05, ^**^p < 0.01, and ^***^p < 0.001. Asterisk(s) over a line are compared with those 2 data bars.

We used immunohistochemistry and confocal microscopy to determine the changes in the myelin basic protein (MBP) in the hippocampal perforant path, which is the principal source of cortical input to the hippocampal formation (Figure 8A). Significant differences among animal groups were observed for the number (*F*_4,75_ = 4.148, *p* = 0.005) and size (*F*_4,75_ = 37.919, *p* = 0.001) of MBP profiles. The number and size of MBP profiles did not change in WT mice with MI or 3×Tg mice. A combination of MI in 3×Tg mice increased (*p* = 0.002) the number but decreased (*p* = 0.001) the size of myelinated axons, indicating uneven demyelination (Figures 8B,C; Tg + MI vs. Tg). Western blots demonstrated the downregulation (*p* = 0.031) of MBP in the dorsal hippocampus of 3×Tg mice with microinfarcts (Figure 8E). DCPLA-ME prevented demyelination in 3×Tg mice with MI (Figures 8B–E).

## Discussion

The present study demonstrated the effect of common carotid artery injection with microbeads that traveled via Circle of Willis and then entered to the right and left brains to induce microinfarcts in several regions of the whole brains. The common carotid artery injection of microbeads is well documented in the literature to induce multifocal microinfarcts in the brains (Silasi et al., 2015; Wang et al., 2017; Shih et al., 2018; Lecordier et al., 2021; Georgakopoulou et al., 2023). In autopsied human brains; cortical, subcortical, or mixed (the whole brain) microinfarcts contribute to pathogenesis of dementia and cognitive disorders (Arvanitakis et al., 2011; Smith et al., 2012). Therefore, it seems likely that microinfarcts outside the hippocampus may directly disrupt important memory networks from several brain regions to the hippocampus (Kitamura et al., 2017).

There are some limitations in the present study. We did not examine change in cerebral amyloid angiopathy. An increase in amyloid deposit around arteries and arterioles is associated with microinfarcts (Kövari et al., 2013; Agrawal et al., 2021; Blevins et al., 2021).

Current evidence supports that although blood flow is further reduced during early stage sporadic AD in human patients, capillary loss in AD brains is not different from capillary loss in age-matched control brains (Brown and Thore, 2011; Hunter et al., 2012). This indicates that sporadic AD does not enhance capillary loss that is already induced by aging. Nevertheless, our study showed that capillary loss was accelerated in middle-aged 3×Tg-AD mice. Capillary loss was also found and shown to progress with age in several parts of the 3×Tg mouse brain, including the hippocampal CA1 area (Quintana et al., 2021). Capillary loss was also found in Tg2576-AD mice (Zhang et al., 2019).

In 3×Tg mice without cerebral microinfarcts, capillary loss was related to aging or hypoxia that increased HIF-1α stability. This result is supported by previous studies showing that HIF-1α is increased in microvessels from AD mice, including 3×Tg (Grammas et al., 2011; Jung et al., 2023). The increase in HIF-1α reduced PGC-1α and c-Myc activities and subsequently reduced TFAM. TFAM is required for replication, transcription, and maintenance of mitochondrial biogenesis (oxidative phosphorylation) and function as well as ROS detoxification (MnSOD, catalase, uncoupling protein 2, peroxiredoxin 3 and 5, thioredoxin 2, and thioredoxin reductase). Therefore, TFAM reduction may lead to mild oxidative stress (Yeo, 2019; Rius-Pérez et al., 2020; Nishigaki et al., 2022). The reduction of PGC-1α and c-Myc activity may also elicit the loss of a multifunctional transcription factor that drives the multiple synthesis functions important for cell division, including VEGF (Baudino et al., 2002; Florea et al., 2013), resulting in capillary loss. Therefore, our results show that in 3×Tg mice, the AD pathogenesis premature aging is more pronounced in capillaries than in arterioles. Our results are in agreement with previous studies showing that patients with AD and Tg2576-AD mice have lower levels of VEGF and PKCε expression in the hippocampal microvessels (Provias and Jeynes, 2014; Millien et al., 2022).

In the 3×Tg mice with cerebral microinfarcts, the results indicate sustained hypoxia increases HIF-1α, TFAM, VEGF, and PKCε in capillaries as well as arterioles in the hippocampal CA1 stratum radiatum. However, mitochondrial MnSOD did not increase in capillaries and arterioles, suggesting that the effect of an age-related decrease in MnSOD is stronger than the effect of sustained hypoxia.

MI induced strong oxidative stress and apoptosis in arterioles but not capillaries in 3×Tg mice. AD-accelerating capillary loss in 3×Tg mice may induce hypoxic preconditioning and brain vascular protection against new hypoxia (Gustavsson et al., 2007; Jarrard et al., 2021). MI increases VEGF in capillaries, resulting in capillary genesis (angiogenesis). Although MI increases capillary density to the WT control level, the capillaries do not function normally due to a decrease in MnSOD that may increase oxidative stress. This results in degeneration of tissue surrounding capillaries and perivascular space dilation, which is closely related with cerebrovascular disease, hemorrhage, and learning and memory defects (Kalaria and Ballard, 1999; Kövari et al., 2013). The present study demonstrates that MI increases VEGF and its downstream VEGF cascade PKCε and ERK1/2. ERK1/2 may increase DNA synthesis and cell over-proliferation in arteriolar wall cells (Cai et al., 2006; Rask-Madsen and King, 2008). Expression of VEGF and PKCε proteins and/or mRNA was previously demonstrated in isolated microvessels from cerebral cortex (Hoehn et al., 2002; Fleegal et al., 2005; Luo et al., 2012; Bai et al., 2015). Chronic hypoxia increases HIF-1α, VEGF, PKCε and BBB leakage in primary cultures of isolated microvessels (Fleegal et al., 2005; Grammas et al., 2011; Luo et al., 2012). Moreover, an increase in VEGF mRNAs in prefrontal cortex as well as VEGF and PKCε proteins in hippocampal CA1 capillaries and arterioles is evident (Moore et al., 2020; Wang H. et al., 2023) in ApoE4-carrier AD human brains that related with increased microinfarcts (Yip et al., 2005).

The increase in arteriolar wall cells induced by MI may reflect MV dysplasia, which is atypical hyperplasia with an increase in immature cells (with different size and morphology) (Fan and Yang, 2007; Su et al., 2008). During the disease process in cases such as brain trauma, ischemia, or inflammation, local angiogenic factors (e.g., growth factors such as VEGF, cytokines, and chemokines) are greatly increased (Fan and Yang, 2007). These angiogenic mediators initially activate focal angiogenesis in the body, including brain tissue, and normal angiogenesis then progresses to MV dysplasia (Su et al., 2008). The change in arteriolar walls does not look like hyperplastic arteriolosclerosis with markedly thickened walls due to cell proliferation and infiltration of lymphocytes in the tunica intima and concentric ring of smooth muscles in the thickened tunica media (Blevins et al., 2021).

Cell-increasing arteriolar wall thickening can be found in non-AD, aged human hippocampus that is correlated with an increase in solid cerebral microinfarcts (Sen and Hongpaisan, 2018). In autopsy-confirmed human brains with cell-increasing arteriolar walls, an increase was found in perivascular space dilation as well as lacunar microinfarcts and infarcts (Sen and Hongpaisan, 2018). Our results confirm that cerebral microinfarcts induced by MI can induce cell-increasing arteriolar walls and perivascular space dilation. Arteriolar wall alteration can reduce blood flow in the capillaries, resulting in hypoperfusion and cortical and subcortical microinfarcts, which appear to be the most robust substrates of cognitive impairment (Kalaria, 2012, 2016; Arvanitakis et al., 2017). Our data reveal that MI complexed with AD accelerates a change in astrocytes that also regulates blood flow. These changes result in pericapillary space dilation, axon demyelination, and synaptic loss.

We recently demonstrated that PKCε activates the mRNA-stabilizing protein HuR that prevents MnSOD and VEGF mRNA degradation and promotes their protein synthesis in cultured human brain MV endothelial cells and T2576 mouse AD hippocampus (Millien et al., 2022). In the present study, we further show that the PKCε activator DCPLA-ME can prevent the effect of MI and AD pathogenesis by increasing MnSOD and VEGF in capillaries. In arterioles, DCPLA-ME increases MnSOD that protects the strong oxidative stress, apoptosis, sustained hypoxia, HIF-1α stability, VEGF rise, and cell exaggerated repair. Preventing change in capillaries and arterioles may indirectly protect changes in astrocytes and A-V coupling, demyelination of axons, synapses, and spatial memory. Additionally, PKCε protects against oxidative damage, inflammation, and apoptosis; supports endothelial integrity via tight junctions; and directly promotes synaptogenesis through membrane accumulation of the PSD-95 (Steinberg et al., 2007; Sonobe et al., 2009; Sen et al., 2016).

^18^F-fluorodeoxyglucose positron emission tomography (PET) and arterial spin labeling MRI, which are cheaper than PET and do not involve radioactivity, are used to detect cerebral hypoperfusion and hypometabolism for various neurological disorders, including mild cognitive impairment and AD (Verclytte et al., 2016; Dolui et al., 2020). Therefore, angiography dilation of perivascular space; and/or white matter hyperintensity can be used as the imaging marker for AD.

## Conclusion

In 3×Tg mice with cerebral microinfarcts, sustained hypoxia (increased HIF-1α and VEGF signals) is dominant with arteriolar wall thickening. DCPLA has a protective effect on arteriolar wall alteration, neuro-glial-vascular disruption, axon demyelination, synaptic loss, and memory defect.

## Data availability statement

The raw data supporting the conclusions of this article will be made available by the authors, without undue reservation.

## Ethics statement

The animal study was approved by the Institutional Animal Care & Use Committee (IACUC) at Thomas Jefferson University. The study was conducted in accordance with the local legislation and institutional requirements.

## Author contributions

HW: Conceptualization, Formal analysis, Writing – review & editing. ZZ: Formal analysis, Writing – review & editing. JH: Conceptualization, Formal analysis, Funding acquisition, Methodology, Project administration, Supervision, Writing – original draft, Writing – review & editing.

## Glossary

3′-UTR: 3′-untranslated region
3×Tg: Triple transgenic
AD: Alzheimer’s disease (AD)
ANOVA: Analysis of variance
APP: Amyloid precursor protein
ARE: AU-rich element coupling
A-V: Astrocyte-vascular coupling
CA: Cornu ammonis
c-Myc: Cellular-myelocytomatosis oncogene
DAPI: 4′,6-diamidino-2-phenylindole
DCP: DCPLA-ME
DCPLA: 8-[2-[(2-pentylcyclopropyl)methyl]cyclopropyl]octanoic acid
DCPLA-ME: DCPLA methyl-ester
ELAV: Embryonic lethal, abnormal vision
ERK: Extracellular signal regulated kinase
GFAP: Glial fibrillary acidic protein
Hu: Human antigen
iNOS: Inducible nitric oxide synthase
HIF-1α: Hypoxia inducible factor-1α
M: Molecular weight marker
MBP: Myelin basic protein
MI: Microocclusion
MnSOD: Mn-superoxide dismutase
MRI: Magnetic resonance imaging
MV: Microvascular
NAD^+^: Nicotinamide adenosine dinucleotide
PBS: Phosphate buffer saline
PGC-1β: Peroxisome proliferator-activated receptor-gamma coactivator-1β
PKCε: Protein kinase Cε
PHD: Prolyl hydroxylase
PSEN: Presenilin
N: Nucleus
ROS: Reactive oxygen species
SE: Standard error
SG: Stratum granulosum
SIRT1: Sirtuin 1
SLM: Stratum lacunosum-moleculare
SM: Stratum moleculare
SO: Stratum oriens
SP: Stratum pyramidale
SR: Stratum radiatum
tau: Tubulin associated unit
TFAM: Mitochondrial transcription factor A
Tg: Transgenic
VEGF: Vascular endothelial growth factor-A
VEGFR2: VEGF receptor 2
pVHL: von Hippel–Lindau
WT: Wild-type
